# Eight pearls in *Clostridium difficile* infection clinical management

**DOI:** 10.1186/1471-2334-13-S1-P47

**Published:** 2013-12-16

**Authors:** Gabriel Adrian Popescu, Șerban Benea, Diana Petrache, Diana Niculescu, Dragoş Florea, Ioana Bădicuț, Monica Popoiu, Alexandru Rafila

**Affiliations:** 1 Carol Davila University of Medicine and Pharmacy, Bucharest, Romania; 2 National Institute for Infectious Diseases “Prof. Dr. Matei Balş”, Bucharest, Romania

## Background

The clinical appearance of *Clostridium difficile* infection (CDI) has changed in the last three years in Romania, due to the emergence of the hypervirulent ribotype 027 of *Clostridium difficile*. It is a risk for less than optimal management of this disease, especially the severe CDI with rapidly fatal outcome.

## Case report

An overview of our 2011-2013 experience retrieved several common mistakes of CDI management (e.g. almost 51% of CDI patients received combined therapy with metronidazole and vancomycin, less than 25% colectomies for cases with toxic megacolon, several secondary cases in patients hospitalized in the same room with a non-diagnosed CDI patient). We have chosen to present eight “pearls” that summarize better the difficulties of CDI clinical and microbiological diagnosis, medical and surgical treatment and control infection measures; each of these ideas is exemplified with situations from our practical experience.

## Conclusion

Several common mistakes were identified in the management of CDI, even in a tertiary hospital specialized in infectious diseases. Medical continuous education based on reliable information its needed for improvement of CDI management.

